# Frequency of LTP (Pru p 3) and profilin (Pru p 4) sensitization in 1052 patients referenced to an Imunoallergology Department in Lisbon

**DOI:** 10.1186/2045-7022-3-S3-P59

**Published:** 2013-07-25

**Authors:** PM Silva, L Pestana, AC Costa, MP Barbosa

**Affiliations:** 1 Imunoallergology, Hospital de Santa Maria, Lisbon, Portugal

## Background

Lipid Transfer Proteins (LTP) and profilins are the most important panallergens in the clinical management of patients with allergy to pollen and plant food (mainly tree nuts and fresh fruits) in the Mediterranean countries. Knowledge of the prevalence of sensitization to these allergens in the population would be extremely useful. In this study we analyze the frequency of sensitization to LTP and profilin in our allergy clinic in Lisbon.

## Methods

We performed skin prick tests to Pru p 3 and Pru p 4 components (peach’s LTP and profilin respectively) in all patients referred to our hospital's allergy department in Lisbon during a period of 6 months. Afterwards, anamnesic features of all sensitized patients were assessed.

## Results

We tested 1052 patients (64% female, mean age 34 years) and found 47 (4.5%) Pru p 3 and 42 (4%) Pru p 4 sensitizations. Co-sensitization was present in 7 patients. Polinosis was present in 70% of the LTP-sensitized, 80% of profilin-sensitized and 86% of the co-sensitized groups, whereas plant food allergy was present in 48%, 23% and 71.4% respectively. Cutaneous, respiratory and gastrointestinal manifestations of food allergy were significantly more frequent in the LTP and co-sensitized groups (p<.001).

## Conclusion

We found lower rates of sensitization than in other studies performed in Mediterranean populations. Although 52% of patients were asymptomatic, LTP sensitization, regardless of profilin co-sensitization, was more often associated with plant-food allergy and presented more severe manifestations.

## Disclosure of interest

None declared.

